# Targeting of Perforin Inhibitor into the Brain Parenchyma Via a Prodrug Approach Can Decrease Oxidative Stress and Neuroinflammation and Improve Cell Survival

**DOI:** 10.1007/s12035-020-02045-7

**Published:** 2020-08-05

**Authors:** Janne Tampio, Johanna Huttunen, Ahmed Montaser, Kristiina M. Huttunen

**Affiliations:** School of Pharmacy, Faculty of Health Sciences, University of Eastern Finland, P.O. Box 1627, FI-70211 Kuopio, Finland

**Keywords:** Astrocytes, Brain-targeted drug delivery, L-type amino acid transporter 1 (LAT1), Perforin inhibitor, Prodrug

## Abstract

The cytolytic protein perforin has a crucial role in infections and tumor surveillance. Recently, it has also been associated with many brain diseases, such as neurodegenerative diseases and stroke. Therefore, inhibitors of perforin have attracted interest as novel drug candidates. We have previously reported that converting a perforin inhibitor into an L-type amino acid transporter 1 (LAT1)-utilizing prodrug can improve the compound’s brain drug delivery not only across the blood–brain barrier (BBB) but also into the brain parenchymal cells: neurons, astrocytes, and microglia. The present study evaluated whether the increased uptake into mouse primary cortical astrocytes and subsequently improvements in the cellular bioavailability of this brain-targeted perforin inhibitor prodrug could enhance its pharmacological effects, such as inhibition of production of caspase-3/-7, lipid peroxidation products and prostaglandin E_2_ (PGE_2_) in the lipopolysaccharide (LPS)-induced neuroinflammation mouse model. It was demonstrated that increased brain and cellular drug delivery could improve the ability of perforin inhibitors to elicit their pharmacological effects in the brain at nano- to picomolar levels. Furthermore, the prodrug displayed multifunctional properties since it also inhibited the activity of several key enzymes related to Alzheimer’s disease (AD), such as the β-site amyloid precursor protein (APP) cleaving enzyme 1 (BACE1), acetylcholinesterase (AChE), and most probably also cyclooxygenases (COX) at micromolar concentrations. Therefore, this prodrug is a potential drug candidate for preventing Aβ-accumulation and ACh-depletion in addition to combatting neuroinflammation, oxidative stress, and neural apoptosis within the brain.

**Figure:**
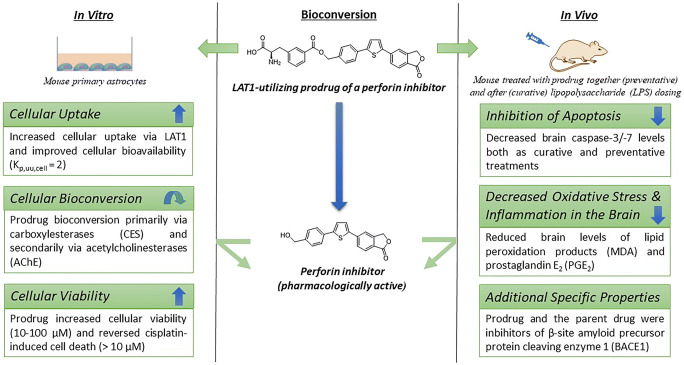
Graphical abstract

Graphical abstract

## Introduction

There is a clear demand for effective drugs to combat central nervous system (CNS) diseases and disorders since CNS disorders represent five of the top-ten causes of long-term disability and account for over 30% of total health care spending [1]. It has also been estimated that every third individual will suffer from a diagnosable CNS disorder during her/his lifetime. Furthermore, these numbers are expected to increase with improved diagnostic techniques and the current demographic trends of an increasingly aging population. Not only do most of the neurodegenerative diseases, such as Parkinson’s (PD), Alzheimer’s (AD), and Huntington’s (HD) diseases, amyotrophic lateral sclerosis (ALS), and multiple sclerosis (MS) lack effective drug therapies, but the same also applies to many other brain disorders, such as stroke, traumatic brain injury, and viral brain infections [1–3]. Despite the huge efforts to reveal the etiologies behind brain diseases, their multifunctional pathologies are still not fully understood [4]. It is recognized that much of the ineffectiveness of CNS drug therapies can be traced from their inability to pass through the blood–brain barrier (BBB) [4, 5] and more importantly to reach their specific target cells within the brain, such as neurons or glial cells [6, 7].

Perforin, discovered in 1975, is a calcium-dependent pore-forming cytolytic effector molecule, which induces apoptosis in its target cells [8]. This glycoprotein is secreted by two cytotoxic effector cells: CD8+ cytotoxic T lymphocytes (CTLs) and natural killer (NK) cells [9, 10]. After target cell recognition, CTLs and NK cells release cytotoxic granules, including perforin, granzymes, and granulysins. Perforin oligomers can form pores on the plasma membrane of the target cell, which permits the entry of granzymes, subsequently inducing apoptosis in the target cells. Perforin can also be transported together with granzymes into the target cell inside endosomes; perforin then disrupts the endosomal membranes within the cells to release apoptosis-inducing granzymes [11]. Therefore, perforin has a crucial role in controlling viral infections as well as in tumor surveillance.

It has been reported that the human perforin allele has single-nucleotide variants with multiple effects on perforin’s activity [12]. Changes in perforin activities can, in turn, result in various conditions, ranging from the very rare but lethal childhood disease familial hemophagocytic lymphohistiocytosis type 2 (FHL 2) [12] to lymphomas [13] and the very common but manageable type 1 diabetes mellitus (T1DM) [14]. Recently, more interest has been focused on brain diseases. It has been reported that perforin has a major role in the axon injury present in the Theiler murine encephalomyelitis virus (TMEV) model of MS [15], in the disruption of the BBB and edema in an experimental cerebral malaria model [16], and in inducible mouse models of seizures and MS [17, 18], as well as in neurotoxicity in an experimental stroke model [19], apoptosis of dopaminergic neurons in a 1-methyl-4-phenyl-1,2,3,6-tetrahydropyridine (MPTP)-induced PD model [20], and β-amyloid internalization and consequent neurotoxicity in neuroblastoma (RA-SH-SY5Y) cells and human primary cortical neurons [21]. Curiously, it has also been found in postmortem brains from patients with MS, AD, and HD that reactive astrocytes contain perforin, not in the granules, but in their cytoplasm around areas of inflammation [22]. This emphasizes perforin’s crucial role also in inflamed brains, as inflammation has been associated indirectly with neurotoxicity in many neurodegenerative diseases as well as stroke and viral infections [23].

**Fig. 1:**
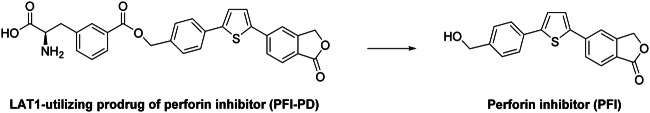
Bioreversible L-type amino acid transporter 1 (LAT1)-utilizing prodrug (PFI-PD) of perforin inhibitor (PFI)

We have previously prepared L-type amino acid transporter (LAT1)-utilizing prodrugs of potent inhibitors of perforin protein [24] and obtained an enhanced and targeted drug delivery across the BBB into the brain [25]. We have also recently reported that by utilizing LAT1, improved intrabrain drug delivery of the parent perforin inhibitor (i.e., cellular uptake and release of the parent drug after enzymatic bioconversion) could be achieved in the neurons, microglia, and astrocytes [26]. Therefore, the present study aimed to evaluate if the increased cellular uptake of the LAT1-utilizing prodrug (PFI-PD) of a perforin inhibitor (PFI) (Fig. 1) could improve also the cellular bioavailability in one of the main target cells within the brain, i.e., mouse primary astrocytes [27]. Moreover, a subsequent aim was to demonstrate for the first time that the improved brain-targeting, target cell-selective uptake, and bioavailability of the released perforin inhibitor can result in enhanced efficacy within the brain, as evaluated with selected biomarkers of inflammation, oxidative stress, and apoptosis both in vitro and in vivo with lipopolysaccharide (LPS)-induced inflammation models. Thus, this study evaluates the feasibility of perforin inhibitors and their brain-targeted prodrugs as multifunctional compounds acting against neurodegenerative diseases, such as AD [28–30].

## Experimentals

### Materials

All reagents and solvents used in analytical studies were commercial and high purity of analytical grade or ultra-gradient HPLC grade. Tris–HCl, ethylenediaminetetraacetic acid–Na_2_ (2 × H_2_O) (EDTA); urea choline chloride; 4-(2-hydroxyethyl)piperazine-1-ethanesulfonic acid (HEPES); MgSO_4_, KH_2_PO_4_, and CaCl_2_ (2 × H_2_O); diclofenac; bovine serum albumin (BSA); lipopolysaccharide (LPS) thiobarbituric acid; butylated hydroxytoluene; malondialdehyde (MDA) tetrabutyl salt; *S*-butyrylthiocholine iodide; 5,5′-dithio(2-nitrobenzoic acid) (DTNB; Ellman’s reagent); and tacrine were purchased from Merck KGaA (Darmstadt, Germany). KCl, NaCl, and NaOH were purchased from J.T. Baker (Deventer, The Netherlands); glucose, formic acid, glacial acetic acid, acetonitrile (ACN), and 2-(*N*-morpholino)ethanesulfonic acid (MES) from VWR International, LCC (Radnor, PA, USA); and dimethyl sulfoxide (DMSO), α-tocopherol, *S*-acetylthiocholine iodide, and donepezil from Acros Organics B.V.B.A (part of Thermo Fisher Scientific Inc., Waltham, MA, USA). Water was purified using a Milli-Q gradient system (Millipore, Milford, MA, USA). The studied LAT1-utilizing prodrug (PFI-PD) and its parent drug (PFI) have been synthesized in-house, and their structural characterization (^1^H NMR, ^13^C NMR, LC-MS) and over 95% purity (elemental analysis) have been confirmed in our earlier publication [24].

### Cell Cultures

Primary astrocytes from the cortex and hippocampi were isolated from 2-day-old adult male mice (C57BL/6JOlaHsd; Jackson Laboratories, Bar Harbor, ME, USA) as previously described [31, 32]. The animals were housed and treated as described below, and cortices and hippocampi were isolated by suspending the brain tissue into the Dulbecco’s Modified Eagle Medium (DMEM; Gibco, ThermoFisher Scientific, Waltham, MA, USA) containing 10% heat-inactivated fetal bovine serum (Gibco, ThermoFisher Scientific, Waltham, MA, USA) and penicillin (50 U/mL)-streptomycin (50 μg/mL) solution (EuroClone S.p.A., Pero, Milan, Italy). The suspension was triturated ten times and thereafter centrifuged at 1500 rpm for 5 min at room temperature. Trypsin-EDTA of 0.25% was added, and the suspension was incubated for 30 min at 37 °C. Fresh culture medium was added, and the suspension was centrifugated at 1500 rpm for 5 min. The astrocytes were cultured in the Dulbecco’s Modified Eagle Medium F-12 Nutrient Mixture (DMEM/F2; Gibco, ThermoFisher Scientific, Waltham, MA, USA) supplemented with l-glutamine (2 mM; EuroClone S.p.A., Pero, Milan, Italy), heat-inactivated fetal bovine serum (10%), penicillin (50 U/mL), and streptomycin (50 μg/mL). The cells were plated on poly-d-lysine–coated flasks in culture medium, and to remove the microglia, the cultures were shaken at 200 rpm for 2 h before the experiments described below were performed. It has been reported earlier that these cultures contain approximately 80% astrocytes (20% microglia) [33]. MCF-7 human breast adenocarcinoma cells (HTB-22; RRID CVCL_0031) were purchased from the European Collection of Authenticated Cell Culture (ECACC, Salisbury, UK, Cat. No. 86012803) and were cultured in standard conditions (37 °C, 5% CO_2_) using DMEM (Gibco, Thermo Fisher Scientific, Waltham, MA, USA) supplemented with l-glutamine (2 mM, Gibco, Thermo Fisher Scientific, Waltham, MA, USA), heat-inactivated fetal bovine serum (10%, Gibco, Thermo Fisher Scientific, Waltham, MA, USA), penicillin (50 U/mL, Gibco, Thermo Fisher Scientific, Waltham, MA, USA), and streptomycin (50 μg/mL, Gibco, Thermo Fisher Scientific, Waltham, MA, USA). All the following studies were carried out as three biological replicates from the different cell passages as well as three technical replicates from the same cell passage. The function of LAT1 was followed between the used cell passages (7-16) with a LAT1 probe substrate, [^14^C]-l-leucine, and noticed to be unaltered.

### Ability to Bind to LAT1

For the transporter-binding studies, the astrocytes were seeded on 24-well plates with a density of 10 × 10^4^ cells/well 3 days before the experiments. After removal of the culture medium, the cells were carefully washed with pre-warmed HBSS (Hank’s balanced salt solution) containing choline chloride (125 mM), KCl (4.8 mM), MgSO_4_ (1.2 mM), KH_2_PO_4_ (1.2 mM), CaCl_2_ (1.3 mM), glucose (5.6 mM), and HEPES (25 mM) adjusted to pH 7.4 with 1 M NaOH. The cells were pre-incubated with 500 μL of pre-warmed HBSS at 37 °C for 5 min before the experiments. To study the ability of studied compounds to inhibit the uptake of a known LAT1 substrate, the cells were incubated at 37 °C for 5 min with an uptake buffer (HBSS, 250 μL) containing 0.76 μM (0.25 mCi/mL or 9.85 MBq/mL) of [^14^C]-L-leucine (PerkinElmer, Waltham, MA, USA) and 0.1–1000 μM of studied compound (or HBSS as blank). After incubation, the uptake was stopped by adding 500 μL of ice-cold HBSS, and the cells were washed two times with ice-cold HBSS (2 × 500 μL). The cells were then lysed with 500 μL of NaOH (0.1 M) for 60 min and the lysate was mixed with 3.5 mL of Emulsifier-Safe cocktail (Ultima Gold, PerkinElmer, Waltham, MA, USA). The radioactivity in the cells was measured by liquid scintillation counting (MicroBeta^2^ counter, PerkinElmer Waltham, MA, USA). Half of the maximum inhibitory concentration (IC_50_) values were calculated by nonlinear regression analysis (fitting the curve to log (concentration) vs. remaining normalized viability).

### Transporter-Mediated Cellular Uptake

For the cell uptake experiments, the astrocytes were seeded on 24-well plates with a density of 10 × 10^4^ cells/well 3 days before the experiments. Cellular uptake of compounds was studied by incubating the cells at 37 °C for 30 min (uptake was linear with all compounds up to 30 min) with compounds at the concentration of 1–200 μM in pre-warmed HBSS buffer (250 μL). Subsequently, the cells were washed three times with ice-cold HBSS and lysed with 250 μL of NaOH (0.1 M) for 60 min. The lysates were diluted with acetonitrile (ACN) including the selected internal standard (diclofenac) with a ratio of 1:3 and centrifuged at 10,000 × *g* for 10 min. The samples were analyzed by liquid chromatography–tandem mass spectrometric (LC-MS/MS) methods described earlier for the PFI (PFI-PD was measured as released parent drug (PFI) since it was completely converted in 0.1 M NaOH to such species) with an Agilent 1200 Series Rapid Resolution LC System together with an Agilent 6410 Triple Quadrupole Mass Spectrometer equipped with an electrospray ionization source by using a Poroshell 120 EC-C-18 column (50 mm × 2.1 mm, 2.7 μm; Agilent Technologies, Santa Clara, CA) [25]. The lower limit of quantification (LLOQ) for the samples was 0.5 nM, and the methods were linear selective, accurate (RSD < 15%), and precise (RSD < 15%) over the range 1.0–100 nM. The concentrations of each compound in cell lysates were calculated from the standard curve that was prepared by spiking known amounts of compounds to ACN including the selected internal standard and normalized with protein concentration. The protein concentrations on each plate were determined as a mean of three samples by Bio-Rad Protein Assay, based on the Bradford dye-binding method, using bovine serum albumin (BSA) as a standard protein and measuring the absorbance (595 nm) by a multiplate reader (EnVision, Perkin Elmer, Inc., Waltham, MA, USA).

### Intracellular Unbound Concentrations

The non-specific binding of compounds (100 μM) was determined in astrocyte homogenate by using the Rapid Equilibrium Dialysis (RED) device (Thermo Fisher Scientific, Inc., Waltham, MA, USA). Briefly, the astrocyte cell suspension (10 × 10^6^ cells/mL) was homogenized with HBSS by the SoniPerp 150 Plus disintegrator (MSE Ltd., London, UK) (2 s × 3). The studied compound was mixed with 100 μL of cell homogenate and added to the reaction chamber. A 350-μL HBSS buffer was added to the buffer chamber. The dialysis plate was incubated at 37 °C for 4 h with shaking. Fifty microliters of samples were taken from the reaction and buffer chambers and equal size of the buffer or blank homogenate were added, respectively, to yield identical matrices. The proteins were precipitated by adding 100 μL of ice-cold ACN (including the selected internal standard; see above), and the supernatants were collected for LC-MS analysis (see above) after centrifugation at 12,000 × *g* for 10 min. The unbound drug fraction (*f*_u,cell_) was calculated by dividing concentration in the buffer chamber by concentration in the reaction chamber and taking into account the effect of homogenate dilution factor (*D* = 45 for 10 × 10^6^ cells/mL cell suspension according to their weight of the cells) as described earlier [34, 35].

The compound concentration ratios in astrocytes (*K*_*p*_) was determined at 0.01–1.0 μM concentrations by comparing the cellular uptake amount (nmol) in cell lysates (0.1 M NaOH) per astrocyte cell volume (0.05810^−15^ L/cell for 10 × 10^4^ cells/well) [36] to the concentrations (nM) detected from the surrounding buffer (HBSS) collected before cell lysing as described previously [34]. The unbound drug concentration ratio (*K*_p,uu_) was then calculated by multiplying the drug concentration ratio (*K*_*p*_) by unbound drug fraction (*f*_u,cell_) [34].

### Ability to Affect Cell Growth

Primary astrocytes or human breast adenocarcinoma cells (MCF-7) were seeded at the density of 20 × 10^3^ cells/well onto collagen-coated 96-well plates. The cells were used for the proliferation experiments 1 day after seeding. The studied compounds (5–400 μM) were added into the growth medium and incubated for 3 days with primary astrocytes (the medium was replaced to a fresh one with studied compounds after daily measurements). With MCF-7 cells, the incubation medium contained 100 μM of cisplatin with or without the studied compounds (10–400 μM). Each day, the cell viability was determined by the resazurin cell proliferation kit (Sigma, St. Louis, MO, USA), which is directly proportional to aerobic respiration and cellular metabolism of cells. The samples were measured fluorometrically by monitoring the increase in fluorescence at *λ*_ex_ 560 nm and *λ*_em_ 590 nm with the Envision plate reader (EnVision, Perkin Elmer, Inc., Waltham, MA, USA). The cell viability was also followed by visualizing the wells with microscopy. The ability of compounds to inhibit the viability of the cells was expressed as percentages (%) compared with the untreated controls.

### Animals

Adult male C57BL/6JOlaHsd mice (Jackson Laboratories, Bar Harbor, ME, USA) were supplied by Envigo (Venray, Netherlands). Mice were housed in stainless steel cages on a 12-h light (07:00–19:00) and 12-h dark (19:00–07:00) cycle at an ambient temperature of 22 ± 1 °C with a relative humidity of 50–60%. All experiments were carried out during the light phase. Tap water and food pellets (Lactamin R36; Lactamin AB, Södertälje, Sweden) were available ad libitum. Neuroinflammation was induced by the treatment with LPS 250 μg/kg i.p once per day for 3 consequent days followed by decapitation on the 4th day. The animals were allocated to one of four treatment groups (*n* = 6 per group): (1) LPS-treated mice, (2) mice treated with LPS and the studied compound (30 μmol/kg; i.v.) for 3 days and 120 min before the decapitation on the 4th day (preventative efficacy), (3) mice treated with LPS for 3 days and with the studied compound (30 μmol/kg; i.v.) only for the last 2 days, and (4) control mice treated with 0.9% NaCl solution i.p. once per day for 3 days. After decapitation, mouse blood and brain samples were collected. The plasma was separated by centrifugation at 1500 × *g* for 6 min. The plasma layer was centrifuged again at 12,000 × *g* to remove the platelets. Plasma was stored at − 80 °C until analysis. The brains were snap-frozen in liquid nitrogen and stored at − 80 °C until analysis.

### Inhibition of Caspase-3/-7 Protein Amount

The effects of the compounds on the enzyme activity of caspase-3 and caspase-7 were evaluated from the snap-frozen mouse brain. Pieces of tissues (*n* = 3) were homogenized in 50 mM Tris–HCl (pH 7.4) 1:10 (*w*/*v*) with a bead mill homogenizer. The homogenate was centrifuged at 10,000 × *g* for 5 min at + 4 °C, and the supernatant was collected for the assay. The enzyme activity was measured with an Apo-ONE Homogenous Caspase-3/7 Assay kit (Promega Corporation, Madison, WI, USA) according to the manufacturer’s instruction. The assay was performed on 96-well plates, each sample having 3 replicates. Each of the sample wells contained 100 μL of the previously collected supernatant and 100 μL of the prepared Apo-ONE reagent. The blank measurement was carried out with the homogenization buffer and Apo-ONE reagent. Once all the samples and reagents were pipetted in the wells, the plate was carefully shaken for 30 s and left for incubation in dark at room temperature. The fluorescence was measured after the incubation with an EnVision 2104 Multilabel Reader (PerkinElmer, Waltham, MA, USA), with *λ*_ex_ 499 nm and *λ*_em_ 521 nm. The measurement was repeated on multiple time points between 0.5 and 24 h.

### Inhibition of Oxidative Stress and Lipid Peroxidation (Malondialdehyde Formation)

Primary astrocytes were seeded at the density of 2 × 10^5^ cells/well onto collagen-coated 6-well plates. The cells were used for the experiments 2 days after seeding, after which they were treated with 0.1 μg/mL LPS in pre-warmed HBSS buffer (250 μL) at 37 °C for 24 h. The control cells were treated with pre-warmed HBSS buffer only. The ability of the studied compounds to inhibit the oxidative stress was evaluated by adding studied compounds (50 μM) in pre-warmed HBSS buffer (250 μL) together with LPS and incubating the cells at 37 °C for 24 h. α-Tocopherol (vitamin E) was used as a positive control. Subsequently, the cells were washed with DPBS (Dulbecco’s phosphate-buffered saline; Gibco, ThermoFisher Scientific, Waltham, MA, USA), detached from the plate by trypsinization (Gibco, ThermoFisher Scientific, Waltham, MA, USA), and centrifuged at 1000 × *g* for 5 min. The supernatant was removed, and the cell pellet was then resuspended with 0.1 M MES buffer (pH 6.0), sonicated for 10 min, centrifuged at 10,000 × *g* for 15 min at 4 °C, and the supernatant was collected. For the brain tissue samples, the supernatant was collected after homogenization as described above (caspase-3/-7 sample preparation) and diluted 1:2 with 50 mM Tris–HCl (pH 7.4).

For the malondialdehyde (MDA) assay, 100 μL of the collected brain tissue supernatants (*n* = 4), 100 μL of 10% (*w*/*v*) trichloroacetic acid (TCA), 75 μL of H_2_O, 5 μL of butylated hydroxytoluene (0.01%), and finally 20 μL of thiobarbituric acid (10 mM) were mixed and incubated in a thermoshaker for 1 h at 95 °C. After incubation, 300 μL of the acetonitrile was added to the mixtures. The mixtures were centrifuged at 10,000 × *g* for 10 min, and supernatants were collected for the UHPLC-UV analysis. The instrument consisted of an Agilent 1290 Infinity II LC System (Agilent Technologies, Santa Clara, CA, USA) combined with an Agilent 1290 Infinity II Diode Array Detector (DAD) (G7117B). The separation of compounds was performed with a Zorbax Eclipse Plus C18 (50 mm × 2.1 mm, 1.8 μm) column. The mobile phase components were water containing 0.1% (*v*/*v*) formic acid (eluent A) and acetonitrile containing 0.1% (*v*/*v*) formic acid (eluent B). The analysis was performed with a following gradient: 0–0.5 min: 10% B, 0.5–2 min: 10% → 25% B, 2–3 min: 25% → 80% B, 3–4.5 min: 80% B, 4.5–5.5 min: 80% → 10% B, 5.5–6 min: 10% B. The flow rate was 1.0 mL/min and the injection volume 1 μL. The column temperature was 25 °C. The UV detector was set at 532 nm. The chromatographic data were collected and compiled with the OpenLAB CDS software version 2.3 (Agilent Technologies, Santa Clara, CA, USA). The amount of malondialdehyde (MDA) in each experiment was quantified from the standard curve; various amounts (1–100 μM) of MDA and thiobarbituric acid in water were incubated in a thermoshaker at 95 °C for 1 h and analyzed simultaneously as duplicates with each studied batch. The results were analyzed as micromole (μmol) of formed MDA (μmol) per milligam of protein (analyzed by Bio-Rad Protein Assay as described above) in the cell assay or as MDA (μmol) per milligram of tissue in the in vivo assay. The lower limit of quantification (LLOQ) for the MDA was 1 μM. The linearity of the calibration curve (1–200 μM), selectivity, accuracy, and precision of the method were suitable. Within-run accuracy and precision of the quality control samples were ±20% of the nominal concentrations.

### Inhibition of Prostaglandin E_2_ (PGE_2_) Production

The effects of the studied compounds to inhibit PGE_2_ production in the mouse brain were evaluated after LPS-induction as curative and preventative treatments. The brain tissue homogenate supernatants (*n* = 4), prepared as described above (caspase-3/-7 sample preparation), were diluted 1:2 with 50 mM Tris–HCl (pH 7.4). The diluted brain supernatant was further diluted 1:5 with an 80% MeOH (*v*/*v*) solution containing the internal standard, PGE_2_-d_4_ (Cayman Chemical Company, Ann Arbor, MI, USA). The dilutions were let to extract at − 80 °C overnight after which they were centrifuged at 14,000 × *g* for 10 min at 4 °C twice before LC-MS/MS analysis. An Agilent 1290 Infinity LC System (Agilent Technologies, Waldbronn, Germany) with a Poroshell 120 EC-C18 column (50 mm × 2.1 mm, 2.7 μm) was used for the liquid chromatography prior to the mass spectrometric analysis. For the MS analysis, an Agilent 6495 triple quadrupole mass spectrometer with an electrospray ionization source (Agilent Technologies, Palo Alto, CA, USA) was used. The LC eluents were water containing 0.1% (*v*/*v*) formic acid (eluent A) and acetonitrile containing 0.1% (*v*/*v*) formic acid (eluent B). The analysis was performed with a following gradient method: 0–5 min: 15% B → 35%, 5–9.5 min: 35% B → 95%, 9.5–12 min: 95% B, 12–12.1 min: 95% B → 15%, 12.1–14 min: 15% B. The flow rate was 0.5 mL/min and the injection volume 5 μL. The column temperature was 40 °C. The mass spectrometric analysis was performed in a negative ion mode with the following parameters: drying gas (nitrogen) temperature was 230 °C, and the drying gas flow rate was 15 L/min, nebulizer gas pressure was 30 psi, sheath gas temperature was 400 °C, and the sheath gas flow was 12 L/min. Capillary voltage was 4500 V. The analyte detection was performed using multiple reaction monitoring with the transitions 351.4 → 315.4 and 351.4 → 271.1 (qualifier) for PGE_2_, and 355.4 → 319.4 and 355.4 → 275.1 for the PGE_2_-d_4_. The fragmentor voltage was 380 V for all the ions, and the collision energies were 9 V, 21 V, 9 V, and 21 V, respectively. The data were acquired using the Agilent MassHunter Workstation software (version B.06.00) and processed with the Quantitative Analysis (B.07.00) software. The lower limit of quantification (LLOQ) for PGE_2_ was 1 nM. The linearity of the calibration curve (1–100 nM), selectivity, accuracy, and precision of the method were validated to be acceptable. Within-run accuracy and precision of the quality control samples were ± 20% of the nominal concentrations.

### Inhibition of Acetylcholinesterase and Butyrylcholinesterase

Inhibitory activities of the studied compounds towards acetylcholinesterase (AChE) and butyrylcholinesterase (BChE) were determined with an endpoint enzymatic assay in mouse brain S9 fraction by the Ellman’s method by using acetylthiocholine (1 mM) for measuring AChE activity and butyrylthiocholine (1 mM) for measuring BChE activity. Briefly, mouse brain S9 fraction was prepared by homogenizing freshly collected mouse brain with 50 mM Tris-buffered saline (TBS) (pH 7.4) (1:4 *w*/*v*), centrifuging the homogenate at 9000 × *g* for 20 min at 4 °C and collecting the supernatant. The supernatant was then diluted 1:10 with phosphate buffer (100 mM; pH 7.0) and mixed with Ellman’s reagent (DTNB; 1 mM) and studied compounds in DMSO (DMSO concentration was less than 0.5%) on a 96-well plate as 3 parallel assays. After reading the absorbance by the Envision plate reader (EnVision, Perkin Elmer, Waltham, MA, USA) at 412 nm, acetylthiocholine or butyrylthiocholine was added and shaken, and the enzymatic activities of AChE or BChE were read at the intervals of 5 min until 30 min. The concentration of studied compounds required to inhibit the specific activity of AChE or BChE (μmol/min/mg protein) was evaluated at a concentration range of 1–800 μM and presented as half of the maximum inhibitory concentrations (IC_50_) for each compound in each biological media at the endpoint (30 min). Unselective ChE inhibitor tacrine and AChE-selective inhibitor donepezil were used as positive controls in both assays.

The detailed type of inhibition of the compounds was evaluated by using 20 μM to 1 mM concentrations of AChE substrate, acetylthiocholine, in the presence of PFI-PD and PFI (8 or 12 μM, respectively; according to their IC_50_ values; Table 1). According to the Hanes–Woolf plots, the ratio of initial substrate concentration to the reaction velocity ([S]/v) was plotted against the substrate concentration (μM). The linear regression was used to calculate *K*_*m*_ value (negative value of *x*-intercept) and *V*_max_ value (1/slope).

### Bioconversion by Carboxylesterases

Bioconversion of prodrug (PFI-PD) into the parent drug (PFI) by carboxylesterases (CES) was determined with human recombinant CES1b and CES2 (Merck KGaA, Darmstadt, Germany) at 37 °C. The incubation mixtures were prepared by mixing the recombinant enzyme (final protein concentration 100 μg/mL) with TBS buffer (pH 7.4) and 5 mM prodrug stock solution in DMSO (the final concentration, the DMSO concentration 2%). The enzyme kinetics was evaluated with several prodrug concentrations from 10 to 100 μM. The reaction mixtures were incubated for 10 min, and the samples (60 μL) were withdrawn at the endpoint. The proteins in the samples were precipitated with ice-cold acetonitrile (60 μL), and the samples were centrifuged for 5 min at 12,000 rpm at room temperature. The supernatants were collected and analyzed (intact prodrug and released parent drug) by the LC-MS/MS method described above [25]. The velocity of produced parent drug in the given time was normalized with the protein amount (μmol/min/mg protein) and plotted against the studied concentration to give Michaelis–Menten kinetic parameters (*V*_max_ and *K*_*m*_) for the enzymatic reaction of each enzyme subtype.

### Inhibition of β-Site Amyloid Precursor Protein Cleaving Enzyme 1 (BACE1)

A fluorometric assay was used to screen the inhibitory effect of the studied compounds on purified human β-site amyloid precursor protein (APP) cleaving enzyme 1 (BACE1) using a fluorescence resonance energy transfer (FRET) peptide technique (SensoLyte© 520 BACE1 Assay kit, AnaSpec, Inc., Fremont, CA, USA) according to the manufacturer’s protocol. Briefly, 1 μM and 10 μM of studied compounds were incubated with the FRET substrate (QXL® 520/ HiLyte™ Fluor 488) and active BACE1 enzyme at room temperature for 30 min. The sequence of the FRET peptide has been derived from the β-secretase cleavage site of β-amyloid precursor protein (APP) with Swedish mutation, which enhances β-secretase to process APP resulting in an early onset of AD. Thus, active β-secretase cleaved the FRET substrate into two separate fragments resulting in the release of HiLyte™ Fluor 488 fluorescence that was measured by the Envision plate reader (EnVision, Perkin Elmer, Waltham, MA, USA) at *λ*_ex_ 490 nm and *λ*_em_ 520 nm. Changes in the amount of this fluorophore caused by the inhibition of β-secretase by the studied compounds (1–10 μM) were compared with the control sample and with a specific inhibitor (0.25 μM provided with the kit (KTEEISEVN-Sta-VAEF-NH_2_) [38]. The results were reported as percentages (%), control sample representing 100% β-secretase activity.

### Data Analysis

All statistical analyses, including Michaelis–Menten, Eadie-Hofstee, and Hanes–Woolf analyses, were performed using the GraphPad Prism v. 5.03 software (GraphPad Software, San Diego, CA, USA). Statistical differences between groups were tested using one-way ANOVA, followed by Tukey’s multiple comparison test and presented as mean ± SD, with statistically significant difference denoted by * *P* < 0.05, ** *P* < 0.01, *** *P* < 0.001.

### Ethical Statement

The experimental procedures involving animals were made in compliance with the European Commission Directives 2010/63/EU and 86/609, and approved by the Institutional Animal Care and Use Committee of the University of Eastern Finland (Animal Usage Plan number ESAVI/3347/04.10.07/2015). All efforts were made to minimize the number of animals used and to minimize their suffering.

## Results

### The Ability of LAT1-Utilizing Prodrug of Perforin Inhibitor to Bind LAT1 in Astrocytes

We have recently characterized mouse primary astrocytes for their LAT1 expression (3.1 ± 0.9 fmol/μg protein) and function ([^14^C]-L-leucine uptake *V*_max_ 2.9 ± 0.4 nmol/min/mg protein; *K*_*m*_ 66 ± 14 μM) [39]. Therefore, the ability of LAT1-utilizing prodrug (PFI-PD) to bind to LAT1 was evaluated as half of the maximal inhibition (IC_50_) of the cellular uptake of [^14^C]-l-leucine (0.76 μM) and compared to the values of the parent perforin inhibitor (PFI) as well as the reported LAT1 inhibitor (KMH-233) [37]. The prodrug was found to bind to LAT1 in astrocytes with a very low, micromolar IC_50_ value (Table 1), while the PFI did not exhibit any interaction with LAT1. Moreover, the ability of the prodrug to bind to LAT1 was considered to be high since it had a 4-time lower IC_50_ value than the reported LAT1 inhibitor (Table 1).

**Table 1:** The ability of perforin inhibitor (PFI), its prodrug (PFI-PD), and LAT1 inhibitor (KMH-233) [37] to bind to LAT1 in mouse primary astrocytes presented as half maximal inhibitory concentrations (IC_50_ values) of LAT1 probe substrate, [^14^C]-l-leucine uptake

Compound	IC_50_ in astrocytes (μM)
PFI	No inhibition
PFI-PD	3.5 ± 1.1
LAT1 inhibitor	14.4 ± 1.2

The data are presented as mean ± SD; *n* = 3–4

### Uptake of LAT1-Utilizing Prodrug of Perforin Inhibitor into Astrocytes

**Fig. 2:**
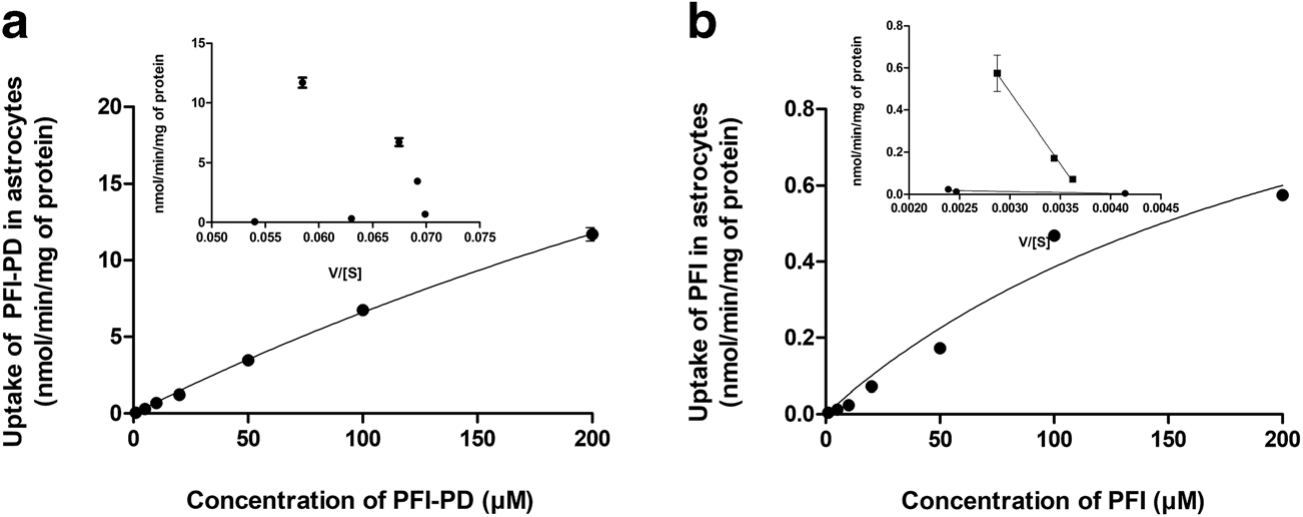
Cellular uptake of LAT1-utilizing prodrug (PFI-PD) (**a**) and its parent drug, perforin inhibitor (PFI) (**b**) into the primary astrocytes over a concentration range of 1–200 μM (mean ± SD, *n* = 3–9), with the Eadie-Hofstee plots shown as insets

Cellular uptake of PFI-PD and PFI into mouse primary astrocytes was concentration dependent (Fig. 2). The maximum transport capacity (*V*_max_) of the prodrug was 54 ± 7 nmol/min/mg protein, and thus, nearly 40 times higher than that of the parent drug (*V*_max_ of 1.4 ± 0.4 nmol/min/mg protein). Curiously, the Eadie-Hofstee analysis revealed that PFI-PD had an autoactivated Eadie-Hofstee profile (Fig. 2a inset). Thus, the prodrug was able to induce LAT1 function or to increase its expression on the plasma membrane [40]. Due to this autoactivation, the uptake profile was almost linear and the kinetic parameter, *V*_max_ and *K*_*m*_ (720 μM), values were relatively high and not reflecting typical LAT1-mediated cellular uptake. The affinity of PFI for its transport mechanism was much lower; the *K*_*m*_ value was 98 μM. Interestingly, PFI was able to utilize two different transport mechanisms accordingly to the Eadie-Hofstee plots (*V*_max_ 0.036 ± 0.007 nmol/min/mg protein and *K*_*m*_ 8.3 μM and *V*_max_ 2.53 ± 0.17 nmol/min/mg protein and *K*_*m*_ 680 μM).

In this study, we also evaluated the intracellular unbound drug concentrations of PFI-PD and PFI since only the free unbound drug concentration can be considered as being pharmacologically relevant when the drug target is located inside the cells, as it is for most of the novel drugs [41]. By utilizing a method reported by Mateus et al., unbound drugs’ accumulation ratios, i.e., *K*_p,uu,cell_ values, can be estimated since this method combines the steady-state intracellular total drug concentration to intracellular binding [34]. In this study, we evaluated the *K*_p,uu,cell_ at 50 nM concentration, which was equivalent to half of the brain total *C*_max_ value (0.12 nmol/g of tissue) and furthermore was the lowest uptake concentration that was able to be detected by the used LC-MS/MS method. The unbound fraction of prodrug (*f*_u_ value) was much lower as compared to its parent drug (Table 2). However, the PFI-PD had better cellular bioavailability (*K*_p,uu,cell_ value of 2.01 ± 0.91) than PFI since the uptake of PFI-PD into the astrocytes was much higher. Despite the higher free unbound fraction, PFI was not detected in cell lysates below 500 nM concentrations.

**Table 2:** Unbound fraction, *f*_u,cell_ (%) and unbound drug accumulation ratios, *K*_p,uu,cell_ values of perforin inhibitor (PFI) and its LAT1-utilizing prodrug (PFI-PD) at 50 nM concentration

Compound	*f*_u,cell_ (%)	*K*_p,uu,cell_
PFI-PD	0.007 ± 0.004	2.01 ± 0.91
PFI	0.53 ± 0.01	n.d.

The data are presented as mean ± SD (*n* = 3)

n.d. = not detected (no compound was detected in cell lysate)

### The Ability of Perforin Inhibitor and its LAT1-Utilizing Prodrug to Affect Cell Growth

**Fig. 3:**
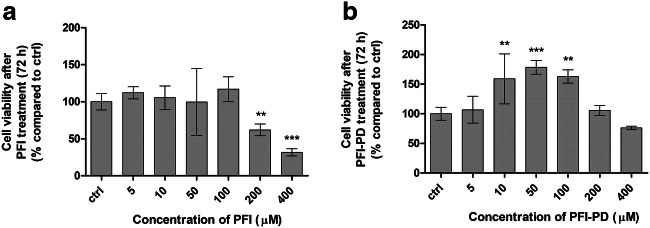
The cell viability of primary astrocytes after 72 h incubation of 5–400 μM perforin inhibitor (PFI) (**a**) and its LAT1-utilizing prodrug (PFI-PD) (**b**) presented as percentages (%) compared to the untreated cells (ctrl) (mean ± SD, *n* = 3–6). An asterisk denotes a statistically significant difference from the respective control (** *P* < 0.01, *** *P* < 0.001, one-way ANOVA, followed by Tukey’s test)

In the present study, the viability of mouse primary astrocytes in the presence of variable concentrations (5–400 μM) of either the PFI or its LAT1-utilizing prodrug was also evaluated by incubating the cells with the compounds for 72 h. PFI had anti-proliferative effects on primary astrocytes with higher concentrations (i.e., over 100 μM), and it inhibited cell growth by 38–68%, while the prodrug did not exert any inhibitory effects on the astrocyte cell growth (Fig. 3). Curiously, PFI-PD increased the astrocyte cell growth at concentrations of 5–100 μM by 35–87% as compared with the control cells.

**Fig. 4:**
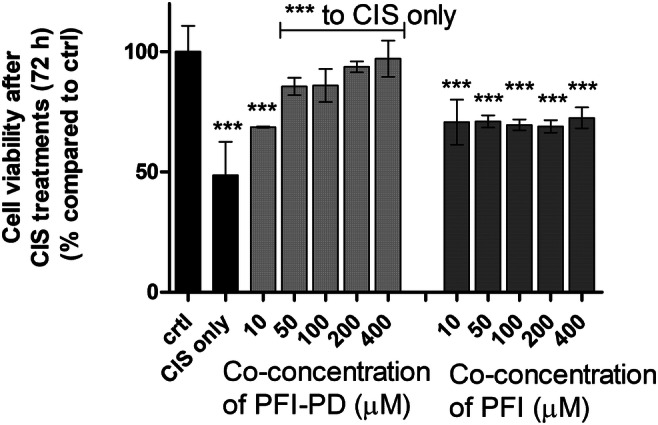
The cell viability of MCF-7 cells after 72 h incubation of cisplatin (100 μM) or co-treatment of 10–400 μM LAT1-utilizing prodrug (PFI-PD) (light gray bars) or perforin inhibitor (PFI) (dark gray bars) together with 100 μM cisplatin. The data are presented as percentages (%) of the treatment groups compared with the untreated cells (ctrl) (mean ± SD, *n* = 3–6). An asterisk denotes a statistically significant difference from the respective control or the cisplatin-induced group (indicated with a separate line) (*** *P* < 0.001, one-way ANOVA, followed by Tukey’s test)

The anti-cancer agent cisplatin is known to induce programmed cell death via caspase (-3, -6, -7, and -8)-dependent and -independent mechanisms [42–44], and since caspase-3 is responsible for apoptosis of neurons and astrocytes [45], we wanted to explore if the LAT1-utilizing prodrug could affect decreased cell viability induced by cisplatin in vitro. Unfortunately, the primary astrocytes were extremely sensitive to the cisplatin treatment even at very low concentrations, and therefore, we used human MCF-7 breast cancer cells to demonstrate the efficacy of studied compounds on caspase-mediated apoptosis. As expected, 100 μM cisplatin inhibited the MCF-7 cell growth by 51% after 72 h incubation (Fig. 4). Interestingly, PFI-PD was able to revert the cisplatin-induced effects on the cells in a concentration-dependent manner. With concentrations of 50 μM or higher, there was no statistical difference in the viability of control cells and cells treated with this compound, while with 50–400 μM concentrations, the cells were significantly more viable as compared with those that were cisplatin-treated. In contrast, PFI was not able to revert the cisplatin-induced effects on cell viability, i.e., there was a significant reduction of cell viability as compared with the control cells.

### The Ability of Perforin Inhibitor and its LAT1-Utilizing Prodrug to Affect Caspase-3/-7 Protein Amount

**Fig. 5:**
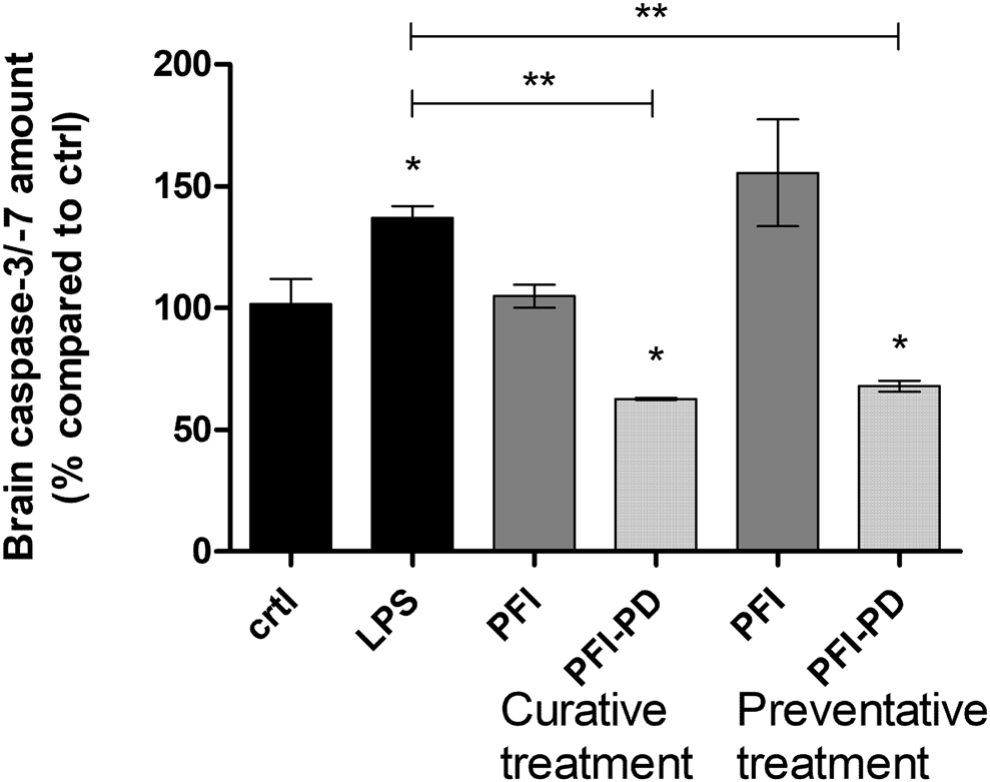
Inhibitory effects of 30 μmol/kg of perforin inhibitor (PFI) (dark gray bars) and its LAT1-utilizing prodrug (PFI-PD) (light gray bars) on caspase-3/-7 levels after lipopolysaccharide (LPS)-induced neuroinflammation administered for 3 consecutive days (250 μg/kg per day). The studied compounds were given as a curative treatment only on the 3rd and 4th days of the experiment or as a preventative treatment every 4 days together with LPS. The data are presented as mean ± SD (*n* = 3). An asterisk denotes a statistically significant difference from the respective LPS or control group (* *P* < 0.05, ** *P* < 0.01, one-way ANOVA, followed by Tukey’s test)

Perforin pore formation on the target cells results in the infiltration of granzymes into the cells and subsequent apoptosis via caspase-dependent and -independent pathways [9]. Therefore, the correlation of improved brain drug delivery of LAT1-utilizing prodrug to caspase-3/-7 levels in LPS-induced mice brain was studied, and compared to treatment with its parent drug. The LPS-model was selected for this study for its simplicity and since it has been reported that immunoregulation and apoptosis in this model are mediated via perforin and caspase-3–dependent pathways [46, 47]. LPS (250 μg/kg) was administered on 3 consecutive days, and the effects of studied compounds were evaluated as a curative treatment, given the compounds (30 μmol/kg) only on days 3 and 4, or as a preventative treatment, with compounds being delivered for 4 days together with 3-day treatment with LPS. Even though the increase of caspase-3/-7 levels by LPS was small (137%), it was statistically significant. Moreover, the treatment with PFI-PD decreased the caspase-3/-7 amounts in both cases, as curative (63%) and as preventative (68%) and notably, below the control level (Fig. 5). The same effect was not seen with the parent drug (105% and 156%, in curative and preventative treatments, respectively).

### The Ability of Perforin Inhibitor and its LAT1-Utilizing Prodrug to Inhibit Oxidative Stress and Lipid Peroxidation

**Fig. 6:**
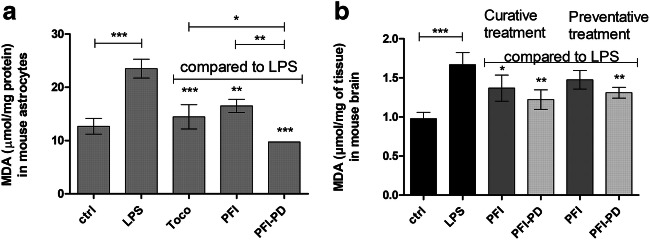
**a** Anti-oxidative effects of 50 μM perforin inhibitor (PFI) and its LAT1-utilizing prodrug (PFI-PD) as well as α-tocopherol (Toco; vitamin E) on lipopolysaccharide (LPS)-induced lipid peroxidation in primary astrocytes after 24 h incubation (in vitro). **b** Anti-oxidative effects of 30 μmol/kg of perforin inhibitor (PFI) (dark gray bars) and its LAT1-utilizing prodrug (PFI-PD) (light gray bars) after lipopolysaccharide (LPS)-induced neuroinflammation (with 250 μg/kg dose per day for 3 consecutive days) in mice (in vivo). The studied compounds were given to mice as a curative treatment only on the 3rd and 4th days of the experiment or as a preventative treatment for 4 days together with LPS. Oxidative stress was measured as malondialdehyde (MDA) formation in the astrocytes (normalized to protein content in **a**) or in the brain (normalized to tissue weight in **b**). The data are presented as mean ± SD (*n* = 3–4). An asterisk denotes a statistically significant difference from the respective LPS or control group (* *P* < 0.05, ** *P* < 0.01, *** *P* < 0.001, one-way ANOVA, followed by Tukey’s test)

As oxidative stress is known to induce caspase-3/-7 production by several pathways, such as via mitochondria or endoplasmic reticulum pathways [48], the anti-oxidative efficacy of the brain-targeted PFI-PD and its parent drug, PFI were examined in LPS-induced (0.1 μg/mL) primary astrocytes as well as LPS-induced mice. As seen in Fig. 6a, both studied compounds (50 μM) were able to inhibit oxidative stress and subsequent lipid peroxidation after 24 h co-incubation with LPS by 30–59% measured as malondialdehyde formation (MDA) in primary astrocytes. Moreover, in these co-treatments, the lipid peroxidation was comparable to the control level (i.e., 12.96 ± 1.47 μmol/mg protein). The same effect was also seen with the positive control, 50 μM α-tocopherol, which was able to inhibit LPS-induced MDA formation by 39%. Thus, the prodrug had a 1.5 times greater inhibitory effect on MDA formation than the known antioxidant α-tocopherol and almost 2.0 times greater effect than its corresponding parent drug.

The brain MDA levels were also measured from the same brain samples as the caspase-3/-7 protein amount. In addition to caspase-3/-7, LPS also increased significantly the lipid peroxidation within the brain (Fig. 6b). Both studied compounds decreased the MDA formation by 31–46%, with PFI-PD showing the highest effect as a curative treatment. However, no statistically significant difference was observed between the compounds.

### The Ability of Perforin Inhibitor and its LAT1-Utilizing Prodrug to Inhibit Brain PGE_2_ Production

**Fig. 7:**
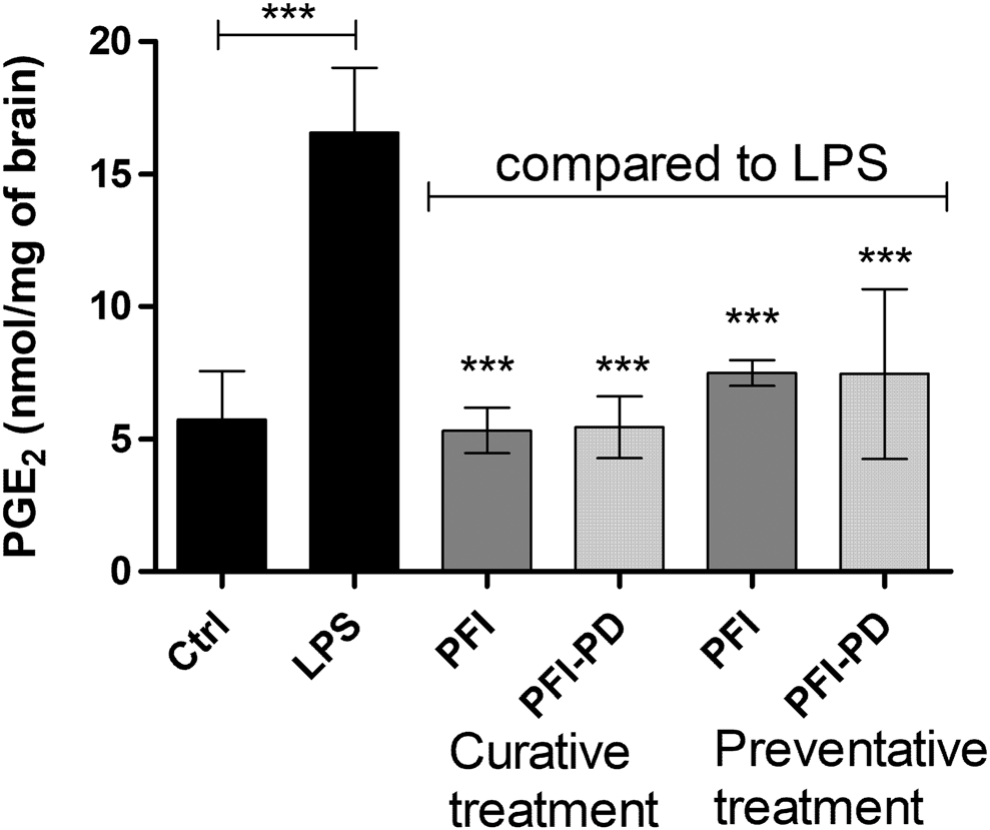
Inhibitory effects of 30 μmol/kg of perforin inhibitor (PFI) (dark gray bars) and its LAT1-utilizing prodrug (PFI-PD) (light gray bars) on prostaglandin E_2_ (PGE_2_) production in the mouse brain after lipopolysaccharide (LPS)-induced neuroinflammation (with 250 μg/kg dose per day for 3 consecutive days). The studied compounds were given to mice as a curative treatment only on the 3rd and 4th days of the experiment or as a preventative treatment for 4 days together with LPS. The data are presented as mean ± SD (*n* = 4). An asterisk denotes a statistically significant difference from the respective LPS or control group (*** *P* < 0.001, one-way ANOVA, followed by Tukey’s test)

Since the LAT1-utilizing brain targeted prodrug was able to inhibit both apoptosis and oxidative stress related to neuroinflammation, we were curious to determine whether it could also affect inflammation markers, and therefore, we measured the PGE_2_ levels from the brain samples. As seen in Fig. 7, LPS increased significantly the brain PGE_2_ production (from 5.72 ± 1.83 to 16.56 ± 2.45 nmol of PGE_2_/mg tissue) due to the induction of cyclooxygenase 2 (COX2) enzyme [49, 50]. Interestingly, the prodrug as well as its parent drug reduced the PGE_2_ production in the brain close to the control levels. This occurred in both curative and preventative treatments (5.32 ± 0.85–7.49 ± 0.48 nmol of PGE_2_/mg tissue), although there was no significant difference between the treatment groups.

### The Ability of Perforin Inhibitor and its LAT1-Utilizing Prodrug to Inhibit AChE/BChE Activity

We have previously reported that the LAT1-utilizing prodrug of ketoprofen is a selective substrate of acetylcholinesterase (AChE) over butyrylcholinesterase (BChE) [51]. Thus, the interactions with AChe and BChE were also evaluated in the present study. Curiously, not only LAT1-utilizing prodrug but also its parent perforin inhibitor was able to inhibit the activity of AChE in mouse brain S9 subcellular fraction with IC_50_ values of 8 and 12 μM, respectively (Table 3). Furthermore, neither of the studied compounds inhibited the activity of BChE. According to the linear regression of Hanes–Woolf plot, the prodrug was concluded to inhibit AChE in a mixed-type manner since the affinity of the enzyme for acetylthiocholine was increased (*K*_*m*_ from 130 to 178 μM), while the velocity of the reaction was decreased (from 2.21 to 2.17 nmol/min/mg protein) (Table 4). Therefore, it was concluded that PFI-PD interacted with AChE with a mixed-type manner, i.e., it is a substrate for AChE, but it can also bind to some site other than the catalytic active site of the enzyme.

**Table 3:** Inhibition of AChE and BChE by perforin inhibitor (PFI) and its LAT1-utilizing prodrug (PFI-PD) in mouse brain S9 subcellular fraction presented as IC_50_ values (mean ± SD, *n* = 3)

Compound (*n* = 3–4)	AChE IC_50_ value (μM)	BChE IC_50_ value (μM)
PFI	12.33 ± 1.50	No inhibition
PFI-PD	8.15 ± 1.32	No inhibition
Donepezil	0.017 ± 0.001	20.74 ± 1.35
Tacrine	0.12 ± 1.10	24.12 ± 1.46

Tacrine and donepezil were used as known positive controls

**Table 4:** Kinetic parameters of enzymatic reaction of AChE inhibited by LAT1-utilizing prodrug (PFI-PD) in mouse brain S9 subcellular fraction and hCES1 and hCES2-mediated bioconversion of PFI-PD (mean ± SD, *n* = 3)

	*V*_max_	*K*_*m*_ (μM)
PFI-PD (12 μM)	nmol/min/mg protein	
AChE	2.21 ± 0.01	130.1 ± 5.1
AChE Inh.	2.17 ± 0.02	178.7 ± 9.1
PFI-PD	μmol/min/mg protein	
CES1b	180.9 ± 36.7	41.8 ± 15.6
CES2	103.2 ± 23.9	8.4 ± 5.8

### Bioconversion of LAT1-Utilizing Prodrug to its Parent Drug Via CES

It has been previously reported that bioconversion of PFI-PD is relatively rapid in the rat liver S9 subcellular fraction (half-life of approximately 55 min) [24], while it is more stable in the mouse brain S9 subcellular fraction (approximately 50% of the prodrug was bioconverted during 5 h incubation) [25] and completely stable in human plasma [24]. However, PFI-PD is bioconverted in primary mouse astrocyte homogenate with a half-life of approximately 6 h [26]. Since CES activity is highest in human and rodent liver, as well as in rodent plasma, although absent in human plasma, it was assumed that CES could be involved in the bioconversion of this prodrug. In this study, we evaluated kinetic parameters for bioconversion of PFI-PD with human recombinant CES1b and CES2. Both CESes were able to convert very effectively the prodrug to its parent drug (Table 4). CES1b had a higher bioconversion capacity but a lower affinity for the prodrug (*V*_max_ 181 μmol/min/mg protein and *K*_*m*_ 42 μM) compared with the CES2 (*V*_max_ 103 μmol/min/mg protein and *K*_*m*_ 8 μM). Thus, it was concluded that it is highly likely that in addition to AChE, CES can also be involved in bioconversion in the brain.

### The Ability of Perforin Inhibitor and its LAT1-Utilizing Prodrug to Inhibit BACE1

**Fig. 8:**
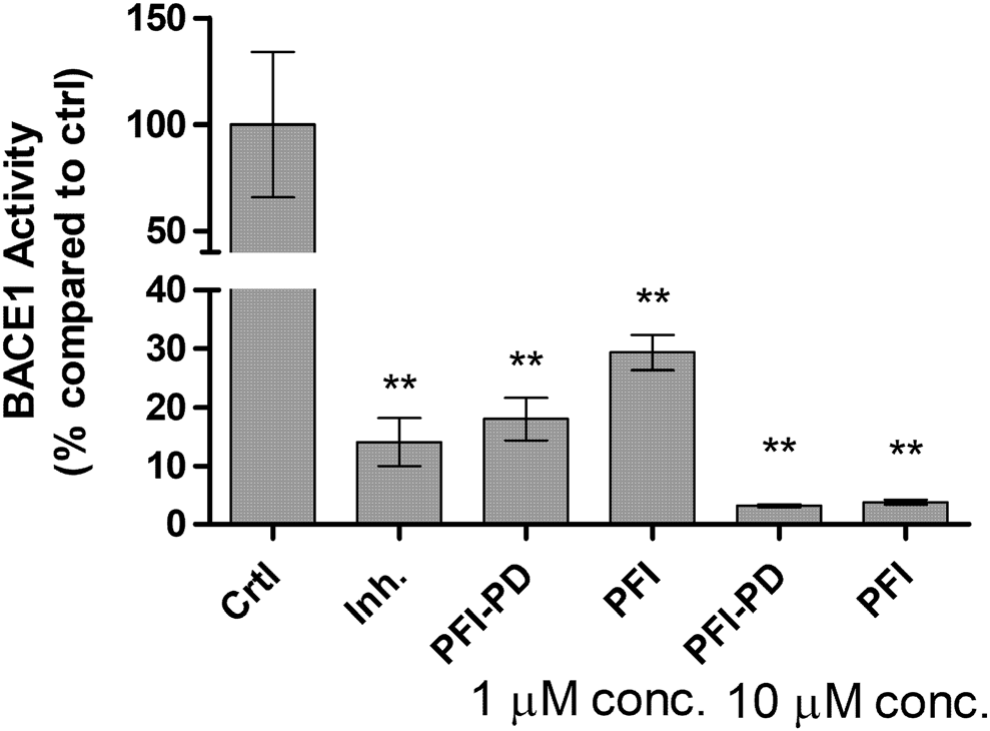
Inhibitory effects of 1 or 10 μM perforin inhibitor (PFI) and its LAT1-utilizing prodrug (PFI-PD) as well as 0.25 μM BACE1 inhibitor (KTEEISEVN-Sta-VAEF-NH_2_) [38] on β-site amyloid precursor protein (APP) cleaving enzyme 1 (BACE1) activity after 30 min incubation. The data are presented as mean ± SD (*n* = 3). An asterisk denotes a statistically significant difference from the respective control (*** *P* < 0.001, one-way ANOVA, followed by Tukey’s test)

We have also previously reported that LAT1-utilizing derivatives of ferulic acid have multifunctional properties that can ameliorate neuroinflammation and oxidative stress [52]. These included inhibition of BACE1 activity. Therefore, the ability of PFI-PD was also evaluated in the present study together with its parent drug and a known BACE1 inhibitor, (KTEEISEVN-Sta-VAEF-NH_2_; provided by the kit) [38]. Unexpectedly, both PFI as well as its LAT1-utilizing prodrug were capable of inhibiting the activity of BACE1 by 71–96% and 82.97%, respectively, at concentrations of 1 and 10 μM, respectively (Fig. 8). In addition, the inhibitory effects were comparable to the known BACE1 inhibitor (0.25 μM; inhibition ca. 86%).

## Discussion

According to the results emerging from this study, it is apparent that the conversion of a perforin inhibitor (PFI) into a LAT1-utilizing prodrug (PFI-PD) can increase the cellular uptake and bioavailability in mouse primary astrocytes. Moreover, we have previously shown that the cellular uptake into the primary astrocytes is LAT1-mediated since the uptake was inhibited by over 70% in the presence of a specific LAT1 inhibitor [26]. The ability of PFI-PD to bind LAT1, measured as the inhibition of the [^14^C]-L-leucine uptake, was relatively high, i.e., the IC_50_ value was 4 μM (Table 1). Moreover, this was in accordance with the value that we have obtained previously in human breast adenocarcinoma cells (MCF-7), 1.54 μM [53]. Noteworthy, the binding ability of this prodrug for LAT1 in astrocytes was much higher than the known LAT1-inhibitor which had an IC_50_ value of only 14 μM [37]. Moreover, the transport capacity of this LAT1-utilizing prodrug into primary astrocytes was many times greater than the one of its parent drug (Fig. 2) since the prodrug was able to autoactivate its LAT1-mediated uptake during 30 min incubation [40]. This mechanism should be examined more thoroughly in the future, as it was not detected during the LAT1-binding studies (5 min). The prodrug had also a reasonable bioavailability in the astrocytes as assessed in the *K*_p,uu,cell_ value (2.01 at 50 nM concentration), at a concentration that reflects the amounts that are distributed to the brain after approximately same dose (*C*_max, brain_ 0.12 nmol/g of tissue, *C*_max, u, brain_ 0.002 nmol/g of tissue) [25], although PFI-PD was highly bound to cellular lipids and proteins (*f*_u,cell_ value of 0.007%) (Table 2). In comparison, the *K*_p,uu,cell_ value could not be calculated for PFI at 50 nM concentration since no compound was detected in cell lysates.

We have recently reported that in addition to astrocytes, LAT1-utilizing prodrugs including PFI-PD can also accumulate more efficiently into the neurons and microglia than a parent drug since these cell types express LAT1 with comparable expression levels on their plasma membrane (3.07–3.77 fmol/μg protein) [26]. One of the most important properties of PFI-PD that was evident in this study was that it did not affect the cell viability of mouse primary astrocytes, while the PFI inhibited cell proliferation at higher concentrations (>200 μM) (Fig. 3). However, these concentrations are over 200 times higher than the IC_50_ value of PFI towards its target protein, perforin, and thus, these concentrations are not clinically relevant [54]. Moreover, in our earlier publication, the bioconversion rate of PFI-PD in astrocyte homogenates has been reported to be very slow, almost 6 h [26]; thus, it is not possible to achieve such a high concentration of released parent drug in the brain that would cause any toxic effects. Together with relatively high unspecific protein/lipid binding of the prodrug in these cells (*f*_u,cell_ = 0.007%), it was not exerting similar antiproliferative effects as its parent drug, even though PFI-PD was transported more effectively into these cells. In turn, PFI-PD was able to induce cell proliferation at lower concentrations (10–100 μM). Even though it may not be closely related, it should be remembered that the prodrug was able to induce LAT1 expression or function at the plasma membrane. This may also increase the uptake of essential amino acids, which may increase cell growth that should be studied more thoroughly in the future [55, 56]. Thus, converting the perforin inhibitor into a LAT1-utilizing and a slow-releasing prodrug can prevent the possible toxic side effects of the parent drug.

Most importantly, PFI-PD was able to retain the cell viability of MCF-7 breast cancer cells in the presence of cisplatin, with prodrug concentrations higher than 50 μM, while PFI did not significantly affect the cisplatin-treated cells (Fig. 4). Since cisplatin induces apoptosis, both via caspase-3/-6/-7/-8–dependent and –independent pathways [42–44], it is highly likely that the effects of the prodrug and/or the released parent drug on cell survival were mediated via direct inhibition of these pathways, without involving the release of cytotoxic caspase-activating granzymes into the cells. Moreover, these cisplatin-reverting effects were concentration dependent, implying that increased cellular drug delivery can result in improved pharmacological effects.

This study also confirmed for the first time that the increased LAT1-mediated brain uptake of perforin inhibitor via prodrug approach, and subsequent accumulation and release of the parent drug in the brain parenchymal cells [25, 26] can result in improved efficacy also in vivo*.* This was demonstrated with the LPS-induced neuroinflammation mouse model, in which the prodrug was able to decrease caspase-3/-7 amounts in the brain more effectively than its parent drug (Fig. 5). According to the earlier pharmacokinetic study with the approximately same dose (23 μmol/kg) [25], the brain *C*_max_ value of PFI-PD was 0.12 nmol/g of tissue, and the corresponding unbound *C*_max,u_ value was 0.002 nmol/g of tissue, while the levels of PFI were below the detection limit of the LC-MS/MS method. Moreover, the amount of released PFI in the brain after the prodrug treatment was 0.01 nmol/g of tissue, and as a free fraction of 0.004 pmol/g of tissue. This implies that the prodrug alone at a nanomolar lever or the released parent drug at a picomolar level was able to reduce the brain caspase-3/-7 levels by over 40% (from 117 to 72–74%). However, it will need to be confirmed in more detail in the future whether these effects of PFI-PD and/or released PFI are attributable to the direct inhibition of caspase-mediated pathways, or are the cytotoxic effector cells (CTL and NK cells) and other caspase-inhibiting mechanisms, such as those mediated via granzymes. However, irrespective of the mechanism, it is known that caspase-3 is responsible for cell death of neurons and astrocytes [45], and therefore, LAT1-utilizing prodrug of PFI has a significant potential to inhibit caspase-mediated apoptosis of neurons and astrocytes in the brain [29].

Nevertheless, the effect of PFI or its prodrug on caspase-3/-7 production can be mediated via several different direct or indirect mechanisms. Increased oxidative stress is one indirect mechanism that can induce caspase-3/-7 production [48]. Therefore, in the present study, the effects of these compounds on lipid peroxidation were evaluated both in vitro with mouse primary astrocytes as well as with LPS-induced mice in vivo. These results showed that the prodrug as well as the parent drug can have anti-oxidative properties in vitro regardless of the transport efficacy into the cells (Fig. 6)*.* Therefore, it is also very likely, that the effects of PFI-PD on caspase-3/-7 inhibition stems from some direct or indirect mechanisms other than the reduction of oxidative stress in the brain.

Curiously, both PFI-PD as well as PFI, which was not extensively transported across the BBB or into the target cells; astrocytes, and microglia (Fig. 2) [25], were also able to reduce the brain PGE_2_ levels either when delivered afterwards or simultaneously with the LPS-induction (Fig. 7). Since there was no significant difference between the treatment groups, neither among the compounds nor administration intervals, we hypothesize that this effect may stem from the peripheral effects of the compounds. LPS can induce not only neuroinflammation, but also peripheral inflammation, and it has been reported that peripheral inflammatory factors as well as inflammatory cells, such as mast cells and T-cells, can augment neuroinflammation, as they can infiltrate across the BBB [57, 58]. After entering into the brain these mediators can act directly or via activation of glial cells first to induce and then to sustain chronic neuroinflammation. Therefore, it is likely and highly beneficial that the prodrug can act both in the brain as well as peripherally to reduce the overall brain PGE_2_ level [30]. Moreover, activated T-cells have been reported to express LAT1 [55, 59, 60], and thus, PFI-PD can readily enter into these cells. It is self-evident that the immunological effects of any LAT1-utilizing prodrug should be evaluated very carefully in the early drug development phase.

In addition to inhibiting caspase-3/-7 production, oxidative stress, and inflammation within the brain, PFI-PD was also found to effectively inhibit BACE1 at the micromolar level (Fig. 8). Curiously, PFI was also able to inhibit BACE1 function. Since this study was carried out with relatively short incubation time (30 min), it is likely that the prodrug can inhibit BACE1 in its intact form. In addition, the prodrug was recognized as a substrate of AChE (Table 3); it seemed to bind to some location other than the catalytic active site (CAS) of AChE. It is well known that AChE also has a peripheral anionic site (PAS) located near to the CAS [61]. Therefore, it is highly likely that PFI-PD can also bind not only to the CAS but also to the PAS of AChE. This is indeed highly probable since the parent drug was also able to inhibit AChE with a comparable affinity (12 μM) to the prodrug (8 μM). However, inhibiting the normal function of AChE as a competitive substrate for acetylcholine or as a released parent drug and as an AChE inhibitor can have additional positive effects, particularly in the AD patient’s brain, i.e., an individual suffering from intensified AChE activity [62]. Taking all the results of the present study together, it can be stated that LAT1-utilizing prodrug of perforin inhibitor, is a brain-targeted multifunctional neuroprotective compound, either as an intact prodrug or via the released parent drug.

In addition to AChE, also CES1b and CES2 were able to convert LAT1-utilizing PFI-PD into PFI (Table 4). Even though the results were obtained by pure recombinant enzymes, it can be speculated that according to the lower *K*_*m*_ values and higher *V*_max_ values, that CESes are most likely the main bioconverting enzymes rather than AChE. CES2 has been reported to be expressed on the endothelial cells of the BBB, which in theory could limit the brain uptake of the prodrug [63]. However, it has been estimated that once the LAT1-substrate is transported into the endothelial cells from the bloodstream, it is rapidly carried also across the abluminal (or basolateral) membrane into the interstitial fluids of the brain via LAT1 due to the very high affinity of the substrates for this transporter [64]. Therefore, it is likely that the majority of the LAT1-utilizing prodrugs are bioconverted to their parent drugs in the brain parenchymal cells, where most of the hydrolyzing enzymes, such as CESes, are located, rather than in the BBB endothelial cells [65–68]. Moreover, we have previously demonstrated that the LAT1-utilizing ester prodrugs are bioconverted and release their parent drugs in all brain cell types that express LAT1, including neurons, astrocytes, and microglia [26]. In these cell homogenates, the half-lives of PFI-PD were approximately 4–6 h. Unfortunately, the other subtypes of the CES family and their localization within the brain are still poorly understood. Furthermore, when designing prodrugs to be bioconverted via CESes, it is essential to take into account the high expression of CES1 in the liver and CES2 in the intestine [69]. Moreover, when studying the prodrugs, the species differences should be acknowledged, e.g., due to the different expression profiles of human and rodent bioconverting enzymes throughout the body, it is difficult to make reliable correlations between these species [51, 70].

## Conclusions

In conclusion, the present study demonstrates for the first time that by utilizing LAT1 for improved brain drug delivery of perforin inhibitors as well as a higher accumulation and bioavailability in the brain parenchymal cells, this can result in improved pharmacological effects, such as decreased production of cellular apoptosis mediators, namely caspase-3/-7. Moreover, LAT1-utilizing prodrugs of perforin inhibitors can simultaneously have direct or indirect multifunctional properties, since the studied prodrug was able to decrease also overall oxidative stress and inflammation within the brain and possibly also in the periphery. Furthermore, it also inhibited specific enzymes, such as BACE1 and AChE, either in the intact prodrug form or as a released parent drug. Thus, this study demonstrated that utilizing LAT1 to improve brain drug delivery of perforin inhibitors and subsequently to enhance cell survival as well as to decrease both oxidative stress and inflammation is a feasible method for further drug development of drugs to combat neurodegenerative diseases like AD.
